# Improving Health Outcomes in Women Who Use Traditional Open Fire Cookstoves by Addressing Cooking Behaviors: A Longitudinal Cohort Study

**DOI:** 10.3390/ijerph23050647

**Published:** 2026-05-13

**Authors:** Amy C. Buckenmeyer, Kevin L. Jantzi, Deanna Marriott, Jeri M. Antilla, Vanesa Abad, Emily Barnes, Katelyn Blackmon, Claire E. Burman, Carly Crave, Christina E. Roembke, Tricia Stathakis, Paige A. Takalamingan, Madison M. Wood, Eve Goddard, Beatrice Hunt

**Affiliations:** 1 College of Nursing and Health Professions, Valparaiso University, Valparaiso, IN 46383, USA; vanesa.abad11@gmail.com (V.A.); e.barnes9317@gmail.com (E.B.); katelyn@empowerherindy.com (K.B.); claireelise42@gmail.com (C.E.B.); carly.crave@gmail.com (C.C.); celundy22@gmail.com (C.E.R.); tricia.stathakis@gmail.com (T.S.); paige.takalamingan@childrenscolorado.org (P.A.T.); mmvons21@gmail.com (M.M.W.); 2 School of Nursing, University of Michigan, Ann Arbor, MI 48109, USAjantilla@umich.edu (J.M.A.); 3 College of Arts and Sciences, Valparaiso University, Valparaiso, IN 46383, USA; kevin.jantzi@valpo.edu; 4 College of Literature, Science, and the Arts, University of Michigan, Ann Arbor, MI 48109, USA; evego@umich.edu (E.G.); beatrich@umich.edu (B.H.)

**Keywords:** air pollution, indoor, community-based participatory research, community health action model for participatory behavior change, global health, health behavior, rural health, transtheoretical model

## Abstract

**Highlights:**

**Public health relevance—How does this work relate to a public health issue?**

- Household air pollution from traditional open-fire cooking remains a major public health problem in rural Nicaragua, disproportionately affecting women.
- This longitudinal cohort study evaluated an improved cookstove intervention aimed at changing cooking practices in a rural community.

**Public health significance—Why is this work of significance to public health?**

- This is the first longitudinal cohort study to apply the novel integration of community-based participatory research and behavior change theory through the Community Health Action Model for Participatory Behavior Change (CHAMP-BC).
- Findings support sustainable improvements in household air pollution exposure-related symptoms for up to seven years, which, to our knowledge, represents one of the longest longitudinal improved cookstove studies among women.

**Public health implications—What are the key implications or messages for practitioners, policymakers and/or researchers in public health?**

- Culturally grounded, community-driven behavior change strategies may strengthen the long-term relevance, acceptability, and sustainability of improved cookstove interventions.
- Public health practitioners and researchers should consider participatory, theory-informed implementation approaches, such as CHAMP-BC, when designing household air pollution interventions in low-resource rural settings.

**Abstract:**

Approximately one-third of the global population cooks over open fires in the home, which are responsible for millions of deaths per year globally. The aim of this longitudinal cohort study was to develop, implement, and evaluate an intervention to improve the cooking behaviors of women in a rural Nicaraguan community. The study employed the Community Health Action Model for Participatory Behavior Change (CHAMP-BC) framework to develop a novel approach to improving cooking behaviors and reducing symptoms associated with household air pollution. The study was conducted from October 2007 to March 2018. Community health needs assessments and community forums were conducted. Readiness to change surveys were administered. Pre- and post-test cookstove implementation surveys were conducted. One hundred sixty-seven primary cooks, primarily women (99%), consented to participate in the improved cookstove program and were followed from baseline for up to seven years post-intervention. There were significant improvements in cough (*p* < 0.0001), chest illness (*p* < 0.0001), shortness of breath (*p* < 0.0001), headaches (*p* < 0.0001), and eye irritation (*p* < 0.0001). The novel CHAMP-BC framework enhanced the research approach by empowering women to change their cooking behaviors while providing them with the autonomy and agency necessary for decision-making regarding evidence-based methods to improve their health.

## 1. Introduction

Cooking over an open fire contributes to high levels of household air pollution and to the global burden of disease, particularly in under-resourced countries. In poorly ventilated homes and kitchens, smoke from traditional open fire stoves can exceed acceptable levels of carbon monoxide and small particulate matter (PM 2.5) by 100-fold [1]. Carbon monoxide and PM 2.5 exposures contribute to the global burden of disease. According to the World Health Organization (WHO), household air pollution is the leading environmental contributor to non-communicable diseases [2]. Household air pollution, from solid fuels and kerosene use, is responsible for almost 3.4 million deaths every year, though solid fuels account for a substantial share of both usage and burden of disease, up to 4%, in low-resource settings [1,3]. This is particularly true in rural Nicaragua, where over 90% of women cook over open fire in poorly ventilated kitchens. The aim of this longitudinal cohort study was to change the cooking behaviors of women to decrease self-reported symptoms associated with household air pollution through the use of the novel Community Health Action Model for Participatory Behavior Change (CHAMP-BC) framework, which is an integration of the Transtheoretical Model of Health Behavior Change and community-based participatory research (CBPR). Researchers have called for the use of behavioral change theory as the foundation for cookstove research, but to date, no one has fully implemented a longitudinal program using the integration of behavior change theory and CBPR with cookstoves [4,5].

### 1.1. Background

Household air pollution is the leading contributor to noncommunicable diseases and has been linked to a broad range of adverse outcomes in women and children, including acute respiratory infections, chronic obstructive pulmonary disease, lung cancer, stroke, and ischemic heart disease [1,2]. Foundational studies conducted in Guatemala and other rural biomass-fuel-dependent communities have shown that household air pollution from traditional cookstoves is associated with adverse respiratory health effects in women and children [6,7,8]. These studies are relevant to the present research because they were conducted in communities with exposure patterns similar to those in rural Nicaragua, where households rely heavily on wood fuel and women experience frequent smoke exposure from cooking.

In rural Nicaragua, household air pollution represents a substantial and gendered public health burden. During the study period, World Bank (2013) estimates suggested that household air pollution from solid fuel use contributed to 284,800 to 412,700 cases of acute respiratory infections each year among women older than 30 years [9]. The same report estimated that household air pollution was associated with approximately 45 to 70 COPD-related deaths and 1280 to 2060 incident cases of COPD in women annually [9].

This burden is especially important in rural Nicaragua because reliance on biomass fuel remains widespread, with exposures occurring in poorly ventilated household kitchens. National data indicate that 91.4% of rural households burn wood in inefficient stoves [10], and prior community-based work in the study region found that nearly all women cooked over open fire in poorly ventilated kitchens [11]. Together, these data support the need for interventions that address both stove design and the behavioral and community conditions that influence sustained stove use.

### 1.2. Target Population

Women in rural Nicaragua are disproportionately exposed to household air pollution because of gendered cooking roles, poverty, and limited access to health-promoting resources. In many rural households, women are the primary cooks and therefore bear a significant burden of daily exposure to smoke from woodburning, traditional open fire cookstoves [9]. This exposure is shaped not only by household cooking practices but also by broader social and structural inequities, including poverty, geographic isolation, and limited access to preventive health services and cleaner household technologies [12,13]. Poverty is also an important driver of continued reliance on wood and other polluting household fuels because cleaner fuels and improved cooking technologies are financially inaccessible or inconsistently available in rural settings.

These inequities are particularly relevant in rural Nicaragua, where economic hardship and limited access to health care affect many households. National data indicate that approximately 30% of the population lives in poverty, and 8% lives in extreme poverty, earning less than USD 1.25 per day [10]. In addition, approximately 48% of the population does not have access to health care services [13]. In this context, women may have few realistic options for reducing household air pollution exposure without community-based, affordable, and locally acceptable interventions. In addition, the same economic conditions that limit access to health care may also constrain household fuel choices, reinforcing continued dependence on biomass fuels for cooking.

Accordingly, the target population for this improved cookstove intervention was women aged 18 years and older living in one small rural village in the Rivas Political Department of Nicaragua. This population was selected because women in the study community were the primary cooks in the households and were routinely exposed to open fire smoke from cookstoves in poorly ventilated kitchens. Focusing on this population allowed the study to examine an intervention among those directly affected by household air pollution and those most likely to benefit from changes in cooking behavior and improved cookstove use.

### 1.3. Behavioral and Environmental Risk Factors

Behavioral and environmental risk factors associated with cooking over open fire were identified during the formative community assessment phases of this research, which were conducted from October 2007 through March 2010 with key informants and community members from the study community [11]. These data were collected through key informant interviews, community health needs assessment interviews, community forums, and community readiness surveys, all of which informed the development of the intervention and the conceptual pathway [14] (see Figure S1).

Findings from this formative work suggested that predisposing, reinforcing, and enabling factors shaped cooking behaviors and household smoke exposure. Predisposing factors, or household-level barriers, included families’ inability to finance an improved cookstove and concerns that changing cooking methods might alter the taste of food. Reinforcing factors included cultural cooking traditions and household and community economic conditions. Enabling factors, or structural barriers, included limited local availability and accessibility of affordable improved cookstoves. Within this context, the primary behavioral risk factor was continued cooking over open fire, and the primary environmental risk factor was inadequate kitchen ventilation, including the absence of chimneys for smoke evacuation in poorly ventilated kitchens [11].

These context-specific factors helped establish the rationale for the improved cookstove intervention and informed the subsequent application of the CHAMP-BC framework. Additional details regarding how these factors were identified are provided in the Section 2 and Section 3.

### 1.4. Literature Review

Several foundational studies across the world have demonstrated the efficacy of reducing household air pollution, improving health, and enhancing acceptability of stoves through improved cookstove programs [6,7,15,16]. Prior studies cited in this review generally evaluated outcomes over relatively short follow-up periods, ranging from several months to approximately 18 months, rather than across multiple years [7,15,16]. A systematic review and meta-analysis also documented similar findings [8]. These studies aside, long-term acceptability and sustainability of improved cookstove programs across the world have not been well established, particularly beyond 18 to 24 months of follow-up.

#### 1.4.1. Indoor Air Pollution

Evidence from rural Guatemala and other biomass-fuel-dependent settings shows that improved cookstoves can meaningfully reduce household air pollution, particularly kitchen concentrations of carbon monoxide and fine particulate matter. In the foundational randomized controlled trial conducted in Guatemalan households using wood fuel, Bruce et al. (2004) found that homes using plancha stoves had substantially lower 24 h kitchen carbon monoxide concentrations than homes using traditional open fires, and fine particulate matter levels showed a similar pattern [6]. These findings are especially relevant to the present study because the Guatemalan households were demographically and geographically similar to and shared important exposure characteristics with rural Nicaraguan households, including wood-fuel use, indoor cooking, and routine smoke exposure among women.

This broader pattern has also been supported by review-level evidence. Pope et al. (2017), in a systematic review and meta-analysis of stove and fuel interventions in low- and middle-income countries, found that improved solid-fuel stoves with chimneys were associated with meaningful reductions in kitchen and personal exposures to PM 2.5 and carbon monoxide under real-world conditions [17]. Similarly, Quansah et al. (2017) reported that household air-pollution interventions generally improved average particulate matter and carbon monoxide concentrations in both personal and microenvironmental measurements, although post-intervention levels often remained above WHO guideline values [18]. Together, these studies support the premise that improved cookstoves can reduce indoor air pollution exposure, even when full elimination of household air pollution exposure is not possible in the economic climate of the community.

#### 1.4.2. Improved Health Outcomes

Improved cookstove interventions have also been associated with improved self-reported health and fewer respiratory symptoms among women. In Guatemala, Diaz et al. (2008) evaluated self-rated health among Mayan women participating in an intervention trial and found that women using improved cookstoves were more likely to report that their health had improved over time than women in the comparison group [16]. Similarly, Smith-Sivertsen et al. (2009), in the Randomized Exposure Study of Pollution Indoors and Respiratory Effects (RESPIRE) trial in Guatemala, compared women using traditional open fire stoves with women using plancha stoves with chimneys and found reductions in respiratory symptoms among women in the improved cookstove group during follow-up [7]. Together, these studies support the potential for improved cookstove interventions to reduce health symptoms associated with household air pollution exposure among women in rural biomass-fuel-dependent communities.

#### 1.4.3. Acceptability of Cookstoves

Acceptability and sustained use of improved cookstoves are critical to whether these interventions can produce long-lasting public health benefits. Mukhopadhyay et al. (2012) examined usage and acceptability of advanced cookstoves in India [15]. Two stove models were distributed to participants. Usage was measured by temperature changes in each unit. The Philips stove was used more often and for more time than the Oorja stove [15]. Acceptability was measured using a questionnaire, which confirmed the usage data [15]. In addition, “all users also reported significant benefits of the Philips stove over the traditional cookstove” [15] (p. 9). Although the Mukhopadhyay study appeared promising, there are no data to date to support long-term sustainability of this improved cookstove program beyond the 12-week follow-up period [15]. More broadly, Lewis and Pattanayak (2012) concluded in a systematic review on cookstove adoption that improved cookstove acceptance is influenced by multiple household, social, and economic factors rather than by stove performance alone [19].

Evidence from Central America suggests that user perceptions, cultural fit, and real-world patterns of stove use strongly influence whether improved cookstoves are adopted and consistently used over time. In rural Guatemala, Bielecki and Wingenbach (2014) found that cookstove choices were shaped not only by efficiency but also by social perceptions and the multiple household functions of traditional stoves, including heating, lighting, and social gathering, underscoring the importance of culturally grounded intervention design [20]. In another Guatemalan study, Paulsen et al. (2019) reported high user acceptance of a catalytic biomass cookstove, with participants using the new stove for most cooking events during the trial; however, follow-up occurred over a relatively short time frame—20 months—rather than over multiple years [21].

Related findings have also been reported in Honduras. Garland et al. (2018) examined real-world use of the Envirofit HM-5000 cookstove in rural and urban Honduran households and showed that usage patterns, fuel consumption, and household conditions remained central to understanding the practical success of improved stove interventions [22]. Although studies such as these support the acceptability and feasibility of improved cookstoves in Central American settings, they also illustrate that most prior evaluations have focused on short-term or cross-sectional outcomes rather than sustained use over several years.

Taken together, these studies support the importance of cookstove acceptability while also demonstrating the need for longer-term evaluation. In the context of the present study, “long-term” refers to sustained use and associated outcomes measured over several years rather than over weeks, months, or a single cross-sectional assessment. This distinction is important because the present study followed participants for up to seven years.

Although improved cookstove interventions are not unique and have continued to be the focus of much research, the innovation in the rural Nicaraguan improved cookstove intervention lies in the integration of the theoretical framework and the research methodology used to guide the intervention and resultant improved health outcomes of the cookstove program. Community-based participatory research methods were used to enhance acceptability and sustainability of the improved cookstove intervention to provide long-term improvements in household air pollution and associated health outcomes of women in the community. Community-based participatory research synergistically integrates with the Transtheoretical Model in its implementation. The Transtheoretical Model has been used to guide CBPR in mitigating cardiovascular risk factors [23], but the integration of this model with CBPR methods has not been implemented to date in developing community-driven improved cookstove programs.

### 1.5. Theoretical Framework

Prochaska and DiClemente’s Transtheoretical Model of Health Behavior Change provided the theoretical foundation for the use of community-based participatory methodology in the cookstove program [24]. The Transtheoretical Model originated from Prochaska and DiClemente’s work on integrating behavior change theories in the field of psychotherapy [24]. The Transtheoretical Model has six stages of change. The model also posits ten processes for change. However, in public health, programs are designed to continue into perpetuity in the maintenance stage as the risk of reverting back to former behaviors exists when sustainability measures are not implemented. As such, the termination stage was not included in this cookstove research program, which resulted in the inclusion of five stages of change.

The stages of change constructs are important because they provide the temporal relationship of change, though this temporal relationship is not always linear [25]. The model allows for ebb and flow of progress toward behavior change. The five stages of change are precontemplation, contemplation, preparation, action, and maintenance. Within these stages, individuals apply the processes of change, which helps them to use cognitive, affective, reflective, and evaluative processes to progress through the stages of change and to maintain behavior change. The ten processes of change are consciousness raising, dramatic relief, self-reevaluation, environmental reevaluation, self-liberation, helping relationships, counterconditioning, reinforcement management, stimulus control, and social liberation.

### 1.6. Community-Based Participatory Research

The community-based participatory research (CBPR) process complements the Transtheoretical Model. The W.K. Kellogg Foundation defined CBPR as “a collaborative approach to research that equitably involves all partners in the research process and recognizes the unique strengths that each brings. Community-based participatory research begins with a research topic of importance to the community with the aim of combining knowledge and action for social change to improve community health and eliminate health disparities” [26] (p. 3). Community-based participatory research is a cyclic and iterative process that involves five phases: (a) partnership, (b) assessment, (c) planning, (d) implementation, and (e) evaluation [26,27].

### 1.7. Community Health Action Model for Participatory Behavior Change (CHAMP-BC)

When the Transtheoretical Model and CBPR are integrated into the improved cookstove program theory, the resultant CHAMP-BC framework provides a novel approach to health behavior change (see Figure S2). This novel model provides the foundation for the improved cookstove program theory, which is a set of assumptions that explains how an intervention is expected to contribute to the intended outcomes. Since the CBPR methods are intrinsic to Prochaska and DiClemente’s Transtheoretical Model, the improved cookstove program theory will be described within the context of the novel CHAMP-BC framework [24].

To guide researchers, theories of action were developed from the constructs of the CHAMP-BC framework (see Table 1). Theories of action describe a set of connected propositions to explain how behavior change may lead to improved health outcomes. These theories of action are embedded in the context of each phase below. Each phase of the CHAMP-BC framework will be defined and its application to the improved cookstove intervention will be described in the methods.

This longitudinal cohort study aimed to empower women to change their cooking behaviors to improve their self-reported symptoms associated with household air pollution through the use of the novel CHAMP-BC framework. Researchers aimed to demonstrate that the use of the CHAMP-BC framework enhanced acceptability and sustainability of an evidence-based improved cookstove program.

## 2. Materials and Methods

The novel CHAMP-BC framework guided researchers in establishing partners in the community, performing a community health needs assessment, completing a community forum, planning for action, implementing improved cookstoves, and evaluating the efficacy and sustainability of the improved cookstoves for up to seven years. The model has five cyclic iterative phases, including Partnership–Precontemplation, Assessment–Contemplation, Planning–Preparation, Implementation–Action, and Evaluation–Maintenance.

### 2.1. Partnership–Precontemplation Phase

During the partnership–precontemplation phase in October 2007, existing community partners were engaged through community capacity building, new partners were developed in the community, and key informant interviews were completed with new and established community partners. Community partners included local leaders, health personnel, educators, community members, and local technical resources involved in cookstove planning and implementation.

A lead interpreter was hired for the duration of the study, October 2007–March 2018. The lead interpreter trained additional interpreters. Training sessions were held for new interpreters prior to deploying research teams for face-to-face interviews. Interpreters were from the same political district to maintain dialect but were not from or known to the target population. Interpreters were used during each interview regardless of the interviewer’s fluency in Spanish.

Face-to-face key informant interviews were conducted. Data were collected using a semi-structured key informant interview guide. The guide included items on regional demographics, major illnesses and injuries, perceived causes of illness, morbidity and mortality, water, food, sanitation, community health resources, education, health education, transportation, communication, and community priorities. A summary of all study instruments, including domains, sample items, response formats, and study phase of administration, is provided in Supplementary Table S1.

Data from the key informant interviews were used to develop a semi-structured community health needs assessment tool in partnership with key informants [28]. The subsequent community health needs assessment tool was a brief interviewer-administered instrument that asked community members to identify the most important health concerns affecting the area, the perceived cause or origin of those health concerns, and proposed solutions to mitigate those health concerns (see Supplementary Table S1).

Finally, during the partnership–precontemplation phase, community forums were conducted. Community members collectively decided to develop poetry and sociodramas to further elucidate their community health concerns. Community members often said, “In every Nicaraguan, there is a poet.” Sociodramas are dramatic representations in which community members act out scenarios for the purpose of action-oriented mitigation of community health problems [29]. Community members created several poems and performed two sociodramas. The sociodramas were specific to household air pollution and will be described in the results.

### 2.2. Assessment–Contemplation Phase

After the community forum and upon consultation with the key informants, a community readiness survey was completed via face-to-face interviews in March 2010. The community readiness survey was designed to assess community perspectives related to indoor air pollution across six domains: community efforts, community knowledge of efforts, leadership, community climate, knowledge about the issue, and resources for prevention efforts. The survey included open-ended questions and anchored 1–10 rating prompts and was used to characterize community readiness to address household air pollution.

### 2.3. Planning–Preparation Phase

In the improved cookstove program, it was determined by the locally elected stove committee, which included five of the key informants and one local mason, that the community’s capacity for implementing improved cookstoves would be reached if a maximum of 50 improved cookstoves were constructed during each implementation cohort. Furthermore, the locally elected stove committee committed to assisting the research team as local guides, while distancing themselves from the interviewee, for baseline data collection during each implementation cohort and follow-up evaluations at one-, three-, five-, and seven-year post-implementation throughout the program. As such, improved cookstoves were implemented through a longitudinal cohort design in 2011, 2013, 2015, and 2017. Per community members’ consensus during the community readiness survey, a raffle was held within one month prior to the construction of the improved cookstoves for each of the four cohorts to equitably allocate available cookstoves among interested participants by population distribution in the volcano, center, and lake sectors of the community.

### 2.4. Implementation–Action Phase

During the implementation–action phase, improved cookstoves were built by local masons in the households of the women who were selected during the community raffle in each cohort: 2011, 2013, 2015, and 2017. Community members indicated that it would only be feasible for the local masons to build the improved cookstoves every other year. At the beginning of each cohort’s implementation–action phase, a semi-structured survey was used to gather baseline data from the participants during interviews in their homes. Baseline data were collected using an interviewer-administered household survey adapted for the study context from the literature [30]. The baseline survey included items on household and family characteristics, fuel use and acquisition, fuel drying, literacy and education, occupation, smoking exposure, kitchen and household structure, stove type, smoke-extraction characteristics, and women’s health and well-being. The health section included open-ended questions regarding perceived effects of smoke from the fire and prompted symptom items related to eyes, cough, chest illness, shortness of breath, and headache. Individuals entered the implementation–action phase 30 days after the completion of their improved cookstoves to allow the construction materials, specifically concrete, to cure. The implementation–action phase commenced when women began to consistently and correctly use their new, improved cookstoves [11].

### 2.5. Evaluation–Maintenance Phase

In the improved cookstove program, the evaluation–maintenance phase was assessed using an interviewer-administered follow-up household survey adapted from the baseline instrument for longitudinal assessment. The follow-up survey retained core items related to fuel practices, smoking exposure, and women’s and children’s health and well-being, including the same prompted symptom items related to eyes, cough, chest illness, shortness of breath, and headache. The follow-up instrument also included questions related to stove satisfaction, perceived wood use, stove maintenance, chimney maintenance, proper use, and the condition of the chimney and plancha. The behavioral maintenance items asked participants: (a) when cooking with one pot, what they did with the other burner; (b) what they did with both burners after cooking; and (c) how they maintained the cookstove, chimney, and plancha. These questions measured progress toward the intermediate behavioral objective of self-efficacy in consistently and correctly using the improved cookstoves (see Figure S2). The evaluation–maintenance phase was evaluated in even years with post-implementation surveys throughout the duration of the research program in March 2012, 2014, 2016, and 2018.

Finally, the evaluation–maintenance phase was measured in March 2018 in all four cohorts with the one-year post-implementation survey (2017 cohort) and the three-year (2015 cohort), five-year (2013 cohort), and seven-year (2011 cohort) post-implementation survey adapted from the baseline and one-year post-implementation surveys. These data measured long-term symptoms in the target population associated with cooking over open fire and sustainability of the improved cookstove program to better inform the integration of the theoretical and methodological frameworks in the novel CHAMP-BC framework (see Figure S2).

### 2.6. Data Collection and Analysis Plan

A convenience sample was recruited during home visits from October 2007 to March 2010 and subsequently self-selected into the improved cookstove research program, where they were randomly selected by population distribution into groups (volcano, center, and lake sectors) during community raffles held in February 2011, 2013, 2015, and 2017. Data were collected through personal, face-to-face interviewing techniques by the research team through trained interpreters during home visits and the community raffles. Quantitative data were collected and analyzed. Demographic information was analyzed using descriptive statistical techniques with SPSS (version 30.0.0.0). To determine if there was a difference in symptoms between the pre- and post-implementation of the improved cookstoves, the Cochran–Armitage Trend Test was performed on complete cases using R software (version 4.4.2) to analyze symptom differences among T0, T1, T2, T3, and T4. This statistical test assesses linear trends in binomial proportions across the levels of an ordinal variable and is the non-parametric equivalent of linear regression.

The longitudinal cohort study is reported according to the Strengthening the Reporting of Observational Studies in Epidemiology (STROBE) cohort study guidelines. As was common in 2007, the study was not registered. The research protocol for the improved cookstove program received approval from the Institutional Review Board (Valparaiso University #16-059) and informed consent was verbally obtained from all participants. The data were de-identified prior to analysis. For formative assessment phases, results are reported as original summary findings that informed intervention development, while a summary of all study instruments, including domains, sample items, response formats, and study phase of administration, is provided in Supplementary Table S1.

## 3. Results

Results will be described in accordance with the CHAMP-BC framework to illustrate the efficacy of the model in cooking behavior change in one small community in rural Nicaragua.

### 3.1. Partnership–Precontemplation Phase

In the partnership–precontemplation phase, 13 key informants consented to interviews in October 2007. Key informants were 31 to 52 years of age, included 7 men and 6 women, and represented a range of educational and occupational backgrounds (see Supplementary Table S2). Interviews were guided by a structured key informant interview guide that asked about community demographics, common illnesses and injuries, perceived causes of illness, morbidity and mortality, water, food, sanitation, health resources, education, transportation, and community priorities. These interviews served to build trust in the community among key leaders and academic partners and to inform the subsequent community health needs assessment.

During the interviews, key informants often identified more than one health concern. Therefore, all health concerns mentioned were recorded as responses rather than limiting participants to a single priority concern. Across the 13 interviews, 39 total health-concern responses were recorded. The most frequent health concerns identified by the key informants were gastrointestinal illnesses (28%, *n* = 11), respiratory illnesses (25%, *n* = 10), urinary tract infections (13%, *n* = 5), and skin infections (13%, *n* = 5). Additional concerns included hypertension (5%, *n* = 2), pregnancy-related concerns (5%, *n* = 2), domestic violence (5%, *n* = 2), diabetes (3%, *n* = 1), and arthritis (3%, *n* = 1; see Supplementary Table S3).

The community health needs assessment began in March 2008 and included 77 individuals from 47 households who ranged in age from 18 to 90 years; participants included 26 men and 51 women and represented a range of educational and occupational backgrounds (Supplementary Table S4). This interviewer-administered tool asked respondents to identify the most important health concerns affecting the area, the perceived cause of those health concerns, and proposed solutions. Similar to the key informant interviews, community members often identified more than one health concern; therefore, all responses were recorded to represent the range of perceived health problems in the community. Across these interviews, 186 total health-concern responses were recorded. The most frequent concerns were respiratory illnesses (35%, *n* = 65), gastrointestinal illnesses (16%, *n* = 30), urinary tract infections (13%, *n* = 24), and fever (9%, *n* = 17) (Supplementary Table S5). Community members identified household air pollution and water and sanitation quality as the primary causes of their health concerns, respectively [11]. From these early formative assessments, we report here the original summary findings that informed the subsequent community forum and intervention development. The community partnership–precontemplation phase aided in building rapport in the community among community members and academic partners.

Results from the community health assessment were shared with community members during a community forum attended by 32 individuals—11 men and 21 women—in March 2010. During the forum, several participants linked cooking over open fire to respiratory symptoms and other health concerns and demonstrated these perceived associations through sociodramas. In one sociodrama, an older woman portrayed coughing, headache, and eye irritation while cooking over an open fire and then acted out relief of these symptoms when using an improved cookstove with a chimney.

These sociodramas prompted discussion among community members about household air pollution, its perceived health effects, and possible community resources to address smoke exposure. During these discussions, some participants identified two local masons from the community who had experience building improved cookstoves, while others described limited awareness of locally available mitigation options.

The community forum provided an opportunity for participants to discuss perceived relationships among cooking over open fire, respiratory illness, and other air pollution-related health symptoms. Within the CHAMP-BC framework, these discussions were interpreted as consistent with increased community awareness of household air pollution as a health concern. Many women expressed concern about the effects of traditional cooking methods on their health. Within the CHAMP-BC framework, these discussions were interpreted as reflecting the process of dramatic relief. These testimonials illustrated the social and practical constraints affecting women’s ability to change cooking practices. Within the CHAMP-BC framework, the discussion was interpreted as consistent with environmental reevaluation and community concern regarding the feasibility of behavior change (see Table 1).

### 3.2. Assessment–Contemplation Phase

In the assessment–contemplation phase, community readiness surveys were conducted with community members in 32 households. Of the community members surveyed, 100% indicated that they cook over open fire in enclosed or semi-enclosed kitchens within their homes. The survey results indicated that community members were ready for change related to household air pollution. Community members linked household air pollution to cooking over open fire. The community members stated that cooking over an open fire increased their risk of respiratory disease, headaches, and eye irritation. The community members also stated that they were ready to change their cooking behaviors [11].

During the community readiness survey, interviews were conducted with community members from 32 households to explore cooking practices and perceptions of household air pollution. A consistent theme across interviews was that women routinely cooked over open fire in enclosed or semi-enclosed homes (100%, *n* = 32). Participants commonly described a perceived link between smoke exposure and household air pollution, and many attributed symptoms such as respiratory problems, headaches, and eye irritation to cooking over an open fire [11]. Overall, narratives reflected an emerging readiness for change, with participants expressing interest in modifying cooking behaviors to reduce smoke exposure. Within the CHAMP-BC framework, these responses were interpreted as consistent with self-evaluation and readiness for action rather than as direct measurement of progression to a new stage of change (see Table 1).

During this phase, community members were shown several models of improved cookstoves and ultimately chose a design that was built out of locally sourced cement, rebar, bricks, and sand with a chimney. This design was similar to an improved cookstove that had been constructed previously in a neighboring community. The community members also suggested that families should invest in their improved cookstoves by providing locally sourced bricks and sand for the base of the cookstove and a meal for the mason. As a result, it was determined that research funding would supply the improved plancha (stovetop) and chimney. Finally, the community members reached a consensus that if there was not enough research funding for all the households in the community to receive an improved cookstove, a community raffle would be an appropriate method of equitably distributing the cookstoves among households. The community readiness survey provided information relevant to the immediate outcome of increased community knowledge about the potential health benefits of cooking with an improved cookstove (see Figure S2).

### 3.3. Planning–Preparation Phase

Per community members’ consensus during the community readiness survey, a raffle was held within one month prior to the construction of the improved cookstoves for each of the four cohorts during the planning–preparation phase. Women in the community prepared for action by self-selecting into the raffle to receive an improved cookstove. Then, women were randomly selected based on population distribution (volcano, center, lake sectors) during the raffle to receive an improved cookstove. The raffle created an opportunity for women to self-select into the improved cookstove program. Within the CHAMP-BC framework, this self-selection process was interpreted as consistent with commitment to action, or self-liberation (see Table 1).

Across all four cohorts, 243 households participated in the improved cookstove raffle. Of the 201 households selected, 167 women consented to participate in the improved cookstove program during baseline data collection. When the improved cookstove program ended in March 2018 due to political unrest in the region, there were approximately 42 of the 243 households in the community who had not received an improved cookstove (~17.3% of the households).

### 3.4. Implementation–Action Phase

The implementation–action phase was initiated when women began using their improved cookstoves 30 days after the concrete had cured. Baseline survey data were collected immediately before improved cookstove usage commenced in March 2011, 2013, 2015, and 2017.

#### 3.4.1. Demographic Characteristics

During the implementation–action phase, 100% of the participants (*n* = 201) who committed to action in the planning–preparation phase received an improved cookstove. Of these participants, 83.1% (*n* = 167) completed the baseline survey. Ninety-nine percent of the participants were women who had a mean age of 42.4 years (SD = 16.8); 88% of the participants attended school, with the majority (60.4%) finishing primary school, or sixth grade. Most of the participants identified as housewives (76.7%) with the remaining identifying as farmers (21.3%). None of the participants were smokers (see Table 2).

#### 3.4.2. Descriptive Characteristics of the Participants’ Kitchens

Data were collected on the characteristics of the participants’ houses. Seventy-six percent of the kitchens were attached to the rest of the main house (*n* = 128). Of the participants’ houses, 51.2% (*n* = 86) did not have any holes in the roofs of their kitchens that would provide ventilation, but 83.7% had eave spaces greater than or equal to 10 cm, which would provide an avenue for the smoke from the woodfire to escape from the kitchen. That said, 17.1% (*n* = 29) of those eave spaces were along the walls within the house, allowing for smoke to only escape into the home. Most of the homes had windows and doors, which would also provide ventilation during cooking, especially since most of the participants, 87.5% (*n* = 146), left the kitchen door open while cooking (see Table 3).

#### 3.4.3. Descriptive Characteristics of Fuel Consumption

Data were collected on various types of fuel consumption, especially wood, since 98% of participants used wood for cooking (*n* = 164). Of those participants who used wood for cooking, 85.7% gathered their own wood for consumption. The scarcity of wood was evenly divided among the participants, with 39.5% (*n* = 66) identifying the amount of wood in the community as plentiful and another 39.5% (*n* = 66) stating that the amount of wood in the community was rather scarce. Although it is recommended that if wood is necessary to use, dry wood be used for burning, 63.6% (*n* = 106) of the participants stated that they use green wood usually or occasionally (see Table 4).

In accordance with the CHAMP-BC framework, women’s adoption of improved cookstoves as a replacement for traditional open fire stoves, along with their continued reported use, was interpreted within the CHAMP-BC framework as reflecting counterconditioning, reinforcement management, and stimulus control during the implementation–action stage. Participants described family and community support that helped them sustain correct, consistent use for at least six months. They also reported close community social networks, with interest in improved cookstoves spreading beyond the initial program participants to other households. These patterns were interpreted as evidence of helping relationships and social diffusion processes that may have supported participation in later raffle cohorts (see Table 1).

### 3.5. Evaluation–Maintenance Phase

In the evaluation–maintenance phase, post-intervention surveys were completed on previously constructed cookstoves in even years during the duration of the research program in March 2012, 2014, 2016, and 2018. Improved cookstoves built in 2011 were followed for seven years, those built in 2013 were followed for five years, those built in 2015 were followed for three years, and those built in 2017 were followed for one year to 2018 (see Table 5).

Although the academic and community partners planned to continue post-intervention evaluations for 10 years, civil unrest in Nicaragua impacted the researchers’ travels to the community after April 2018. Though academic partners intended to re-enter the community once the political unrest subsided, unfortunately, COVID-19 began shortly thereafter, which further limited reengagement for an additional 10-year follow-up. Subsequently, academic and community partners concluded that it was imperative to move the novel CHAMP-BC framework forward to publication, which ultimately resulted in making the difficult determination to end the improved cookstove program and any subsequent data collection.

Baseline, one-, three-, five-, and seven-year post-implementation data were analyzed to evaluate progress toward improved health outcomes. Eighty-eight percent (*n* = 147) of the participants were followed up to seven years during the maintenance stage. Loss to follow-up was primarily related to economic conditions and the need for women to leave the community for work in Costa Rica.

The Cochran–Armitage Trend Test indicated that there was a significant difference in the women’s cough (*p* < 0.0001), chest illness (*p* < 0.0001), shortness of breath (*p* < 0.0001), headache (*p* < 0.0001), and eye irritation (*p* < 0.0001) symptoms over time (see Table 6).

Within the CHAMP-BC framework, women’s continued reported use of improved cookstoves without reverting to open-fire cooking for at least one year and up to seven years was interpreted as consistent with self-efficacy in cooking behavior. Over time, women reported improvements in cough, chest illness, shortness of breath, headaches, and eye irritation (see Table 6). Within the CHAMP-BC framework, these sustained patterns of stove use and symptom improvement were interpreted as consistent with maintenance of behavior change.

## 4. Discussion

The findings from this improved cookstove research study are promising and should be interpreted in the context of both prior cookstove studies and the theoretically based, participatory design of the present intervention. Application of the CHAMP-BC framework enhanced the research approach by integrating community-based participatory research with behavior change theory to support locally relevant decision-making regarding stove design, implementation, and sustained use. Participants demonstrated readiness for change, commitment to action, and sustained use of improved cookstoves over time, and these patterns were accompanied by significant improvements in self-reported cough, chest illness, shortness of breath, headache, and eye irritation.

The duration of follow-up in the present study is an important contribution. Among the peer-reviewed improved cookstove studies we identified that followed women’s symptom or symptom-related health outcomes, prior follow-up generally extended from approximately 1 year to 18 months. In the RESPIRE trial in Guatemala, Smith-Sivertsen et al. (2009) reported women’s respiratory symptom outcomes over approximately 12 to 18 months of follow-up [7]. Díaz et al. (2008) reported self-rated health outcomes after a mean of 16.31 months of plancha use among Mayan women in Guatemala [16]. Alexander et al. (2014) evaluated respiratory health-related quality of life in women at 1-year post-intervention in rural Bolivia [31]. In contrast, the present study followed women for up to seven years, allowing examination not only of short-term improvement, but also of the sustainability of reported symptom changes over time.

Accordingly, to our knowledge, this study is among the longest longitudinal improved cookstove studies to report sustained symptom outcomes among women. Although some cookstove studies have reported longer-term follow-up for exposure, stove use, or adoption outcomes, published follow-up of women’s symptom outcomes has generally been substantially shorter. This distinction is important when interpreting the contribution of the present study.

Several features of the present study may help explain the sustainability of the findings. First, the intervention was developed through formative assessment with key informants and community members, allowing local concerns, constraints, and resources to shape the intervention design. Second, community members selected a stove model that was locally acceptable and feasible to construct using local materials and labor. Third, households contributed locally sourced materials and meals for the masons, which may have strengthened investment in and ownership of the intervention. Fourth, local masons and a locally elected stove committee supported implementation, and repeated follow-up measures over multiple cohorts allowed sustained contact with the community over time. Taken together, these features likely strengthened acceptability, feasibility, and accountability in ways that are often difficult to achieve in more top-down cookstove programs. Beyond the cookstove itself, the theoretically based, participatory design of the intervention may help explain the sustainability of the observed findings.

The participatory design of this study is also important considering broader CBPR literature. CBPR has been described as a collaborative approach that can strengthen trust, improve the relevance of interventions to community priorities, and support implementation and sustainability over time. In the present study, formative assessment with community members, local selection of stove design, use of local masons, shared household contributions, and continued community involvement across implementation cohorts may have strengthened acceptability, ownership, and sustained use of the improved cookstoves. Although the present study was not designed to isolate the independent effect of CBPR on symptom outcomes, the durability of the findings is consistent with literature suggesting that participatory approaches may enhance the long-term sustainability of public health interventions [19,32,33,34,35].

Accordingly, we interpret the seven-year findings not simply as evidence of stove efficacy, but as evidence that intervention durability may depend on the combination of appropriate cookstove design, community ownership, and behaviorally informed implementation. Future studies should continue to evaluate not only whether improved cookstoves reduce exposure and symptoms, but also which participatory and behavioral strategies are most effective for sustaining correct and consistent use over multiple years.

The present study also contributes to cookstove research by applying a theory-informed, participatory framework to intervention design and implementation. Because cookstove interventions often face challenges related to adoption and sustained use, the integration of CBPR with behavior change theory may offer a useful approach for strengthening long-term intervention relevance and sustainability in similar settings. Although the present study was not designed to isolate the independent effect of the CHAMP-BC framework, the consistency of the findings suggests that behaviorally informed and community-driven implementation strategies warrant further study in household air pollution research [4,36].

Although the findings from the improved cookstove research are consistent with previous research, it was hypothesized by the researchers that the integration of the Transtheoretical Model with CBPR, resulting in the CHAMP-BC framework, would benefit acceptability of the improved cookstoves and would enhance sustainability of the improved cookstoves. To our knowledge, this study is among the longest longitudinal improved cookstove studies to report sustained symptom outcomes among women.

### Limitations

Although the findings from the improved cookstove research program are promising, the study was not without limitations. The study had potential for both selection and information biases. Since participants self-selected into the raffle, this could present a selection bias if participants in the improved cookstove research program were different from those who decided not to participate. The nature of the convenience sample limits generalizability beyond the target population. Since the participants self-reported symptoms during interviews, the potential for information bias was present. The likelihood that participants reported symptoms because they knew they were exposed is greater than in the general population. That said, this bias possibly posed a nondifferential misclassification since all participants likely had the same exposures and were interviewed using the same measures.

Furthermore, use of a cohort design likely posed a limitation. Since everyone in the same geographical area was exposed, there was no opportunity for comparison with an unexposed group that would have had the same demographic and geographic characteristics. As such, academic–community partners could not account for possible secular changes that would affect the health of these communities. However, baseline data from each improved cookstove recipient served as the within-subject control group. Although the participatory and theory-informed design of the intervention may help explain the durability of the findings, the study was not designed to isolate the independent contribution of the CHAMP-BC framework. Though researchers acknowledge that the inevitable delay in publishing the findings from the improved cookstove program may pose some temporal limitations, it is important to note that the relevance of both the cookstove science and the CHAMP-BC framework is important to disseminate.

## 5. Conclusions

This is the first fully implemented CBPR program founded on the Transtheoretical Model of Health Behavior Change that examined household air pollution and associated health symptoms: When integrated, the CHAMP-BC framework provided a novel approach to targeting cooking behavior change. It is widely recognized that CBPR enhances acceptability and sustainability of interventions [20,21]. It is also recognized that behavior change theory is essential to advancing public health science [25]. This study suggests that a community-driven, theory-informed approach to improved cookstove implementation may support sustained symptom improvement over time. Future research should further evaluate the application of the CHAMP-BC framework and related participatory behavior change approaches in household air pollution interventions, particularly in low-resource settings where long-term adoption remains a challenge.

The CHAMP-BC framework has strong implications for public health program theory, research, and practice. The model provides a generalizable, participatory framework for other community health programs focused on behavior change. The ease with which the model aligns with the method may provide a mechanism to effectively enhance acceptability and sustainability of community health interventions beyond those that address household air pollution.

## Figures and Tables

**Table 1:** Theory of action.

CHAMP-BC Constructs	Theory of Action ^1^
Partnership–Precontemplation Phase	If key informants and community members complete community health needs assessment interviews, the assessments will allow community members to engage in *environmental reevaluation* of health problems in their community. If community forums provide culturally relevant health education, the forums will *raise consciousness*, provide *dramatic relief*, and allow *environmental reevaluation* to occur among community members about health problems in the community.
Assessment–Contemplation Phase	If community readiness surveys are performed, the surveys will allow women to *self-evaluate* their own knowledge of the health risks associated with cooking over open fire and their readiness to change their cooking behavior to an improved cookstove.
Planning–Preparation Phase	If a raffle is held to distribute improved cookstoves, the raffle will allow women to make a commitment to action, called *self-liberation*, and to self-select into the improved cookstove program.
Implementation–Action Phase	If women substitute cooking on their improved cookstoves for traditional open fire stoves, recognize the benefits of doing so, and have the support of *helping relationships* from their families and community, then *counterconditioning*, *reinforcement management*, *and stimulus control* will be achieved when the women maintain consistent and correct use of their improved cookstoves for at least 6 months.
Evaluation–Maintenance Phase	If women continue to use their improved cookstoves consistently and correctly without reverting to cooking over open fire, then the women will actualize *self-efficacy* in their cooking behavior. If the women actualize *self-efficacy* in their cooking behavior, open fire-associated health symptoms of women (≥18 years old) will improve.

^1^ *Processes of Change* integrated from the Transtheoretical Model.

**Table 2:** Demographic characteristics of the participants.

Variable	Responses	*n* or Mean	(% or SD)
Age		42.4	(16.8)
Sex	Male	2	(1)
	Female	165	(99)
Attended School	No	20	(11.7)
	Yes	147	(88.3)
Grade Completed	1–3	36	(21.3)
	4–6	100	(60.4)
	7–9	31	(18.3)
Able to Read	No	20	(11.7)
	Yes	147	(88.3)
Occupation	Housewife	128	(76.7)
	Farmer	36	(21.3)
	Other	3	(2)
Smoker	No	167	(100)
	Yes	0	(0)

**Table 3:** Descriptive characteristics of the participants’ kitchens.

Variable	Responses	*n* or Mean	(% or SD)
Kitchen Type	Separate building	27	(16.2)
	Separate attached	128	(76.7)
	In main living area	12	(7.1)
Roof Ventilation	None	86	(51.2)
	Small holes	51	(30.2)
	Large holes	15	(9.3)
	Open roof	15	(9.3)
Eave Depth	None	8	(4.7)
	<10 cm	19	(11.6)
	10–30 cm	78	(46.5)
	>30 cm	62	(37.2)
Eave Length	None	4	(2.4)
	All around the room	81	(48.8)
	Along outside walls	53	(31.7)
	Along walls of house	29	(17.1)
Windows	None	44	(26.2)
	One	95	(57.1)
	Two	16	(9.5)
	Three	8	(4.8)
	Four	4	(2.4)
Doors	One	84	(50.4)
	Two	63	(37.5)
	Three	20	(12.1)
Door Open while Cooking	No	21	(12.5)
	Yes	146	(87.5)

**Table 4:** Descriptive characteristics of fuel consumption.

Variables	Responses	*n* or Mean	(% or SD)
Fuel for Cooking	Wood	164	(98)
	LPG	3	(2)
Gather/Buy Wood	All gathered	143	(85.7)
	Mostly gathered	12	(7.1)
	Mostly bought	4	(2.4)
	All bought	8	(4.8)
Amount of Wood	Very scarce	13	(7.9)
	Rather scarce	66	(39.5)
	Just enough	22	(13.2)
	Plentiful	66	(39.5)
Use of Green Wood	Never	61	(36.4)
	Occasionally	80	(47.7)
	Usually	26	(15.9)

**Table 5:** Cookstove time.

Cookstove Time				
T0	T1	T2	T3	T4
2011	✓	✓	✓	✓
2013	✓	✓	✓	
2015	✓	✓		
2017	✓			
(*n* = 167)	(*n* = 147)	(*n* = 80)	(*n* = 38)	(*n* = 19)

**Table 6:** Symptoms over time.

	%(*n*) Pre-T0	%(*n*) Post-T1	%(*n*) Post-T2	%(*n*) Post-T3	%(*n*) Post-T4	*p*-Value *
Cough (No)	26%(44)	79%(116)	94%(75)	84%(32)	95%(18)	<0.0001
Cough (Yes)	74%(123)	21%(31)	6%(5)	16%(6)	5%(1)	
Chest Illness (No)	44%(74)	88%(129)	93%(74)	95%(36)	89%(17)	<0.0001
Chest Illness (Yes)	56%(93)	12%(18)	7%(6)	5%(2)	11%(2)	
SOB (No)	41%(69)	88%(129)	91%(73)	87%(33)	84%(16)	<0.0001
SOB (Yes)	59%(98)	12%(18)	9%(7)	13%(5)	16%(3)	
HA (No)	35%(58)	78%(115)	83%(66)	79%(30)	79%(15)	<0.0001
HA (Yes)	65%(109)	22%(32)	17%(14)	21%(8)	21%(4)	
Eyes (No)	18%(30)	82%(121)	94%(75)	76%(29)	89%(17)	<0.0001
Eyes (Yes)	82%(137)	18%(26)	6%(5)	24%(9)	11%(2)	

* *p*-values are based on the Cochran–Armitage Trend Test.

## Data Availability

The data from this study are not publicly available, as participants did not provide consent for sharing beyond the research team.

## Supplementary Materials

The following supporting information can be downloaded at: https://www.mdpi.com/article/10.3390/ijerph23050647/s1, Figure S1: Symptoms among women from cooking over open fire; Figure S2: Community Health Action Model for Participatory Behavior Change (CHAMP-BC): integration of the transtheoretical model with community-based participatory research; Table S1: Summary of survey instruments, domains, sample items, response format, and study phase of administration; Table S2: Characteristics of key informants involved in the partnership-precontemplation phase; Table S3: Key informant interview findings: primary health concerns identified during the partnership-precontemplation phase; Table S4: Characteristics of community health needs assessment participants; Table S5: Community health needs assessment findings: primary health concerns identified during the partnership-precontemplation phase.

## Abbreviations

The following abbreviations are used in this manuscript:

CHAMP-BC	Community Health Action Model for Participatory Behavior Change
CBPR	Community-based participatory research
