# Supplementary material for: Improving Health Outcomes in Women Who Use Traditional Open Fire Cookstoves by Addressing Cooking Behaviors: A Longitudinal Cohort Study
Source: Int J Environ Res Public Health. 2026 May 13;23(5):647. doi: 10.3390/ijerph23050647 (PMC13206588; doi:10.3390/ijerph23050647)
Supplement: Supplementary file 1 [file ijerph-23-00647-s001.zip › IJERPH Supplemental Figures S1 and S2 Final v.3.pdf]

Supplemental Figures

Figure S1

Symptoms among women from cooking over open fire.

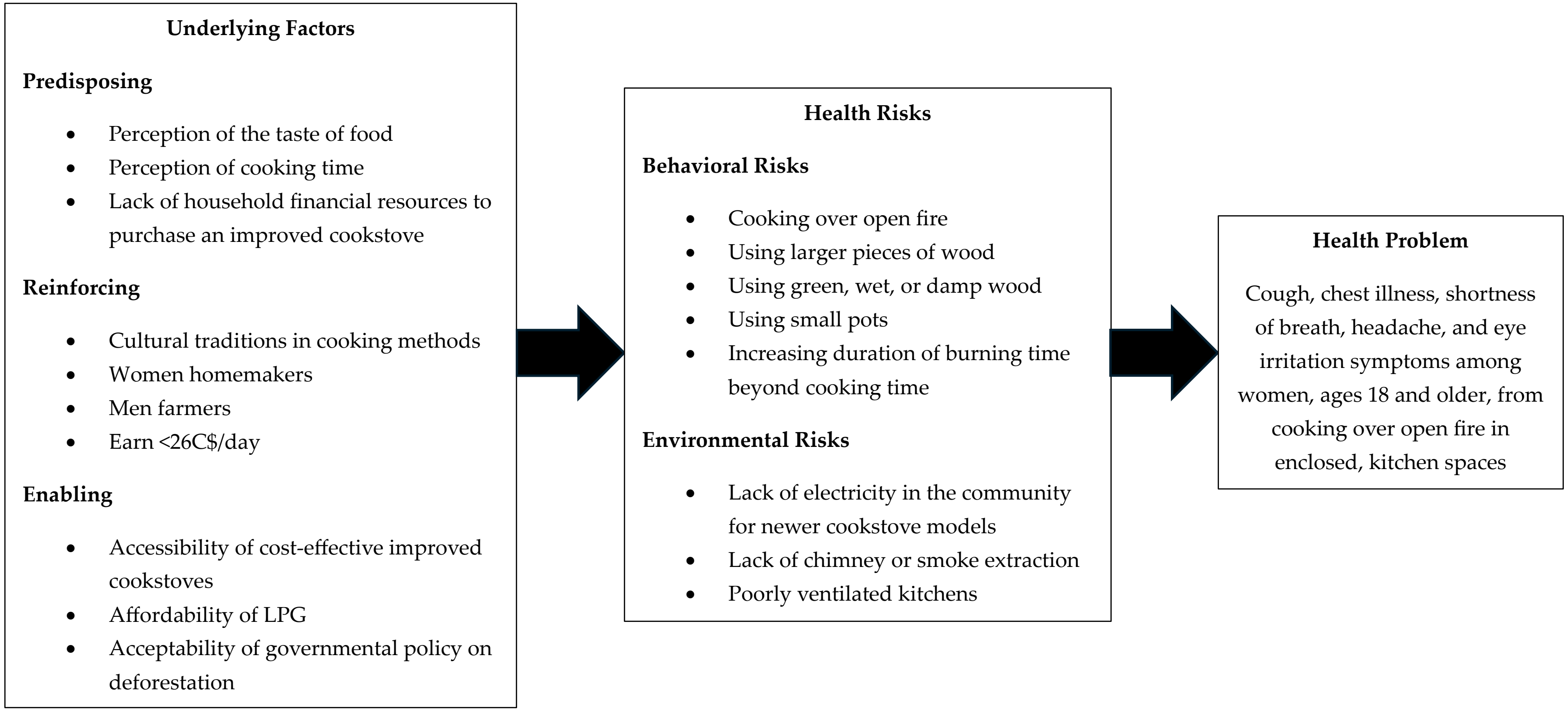

Figure S2

Community Health Action Model for Participatory Behavior Change (or CHAMP-BC): Integration of the Transtheoretical Model with community-based participatory research.

ACTIVITIES	OUTPUTS	IMMEDIATE OUTCOMES
**Partnership-Precontemplation Phase**<br>Survey key informants via interviews.<br>Complete community health needs assessment with community members.<br>Provide culturally relevant, community educational forums.	**Partnership-Precontemplation Phase**<br># key informants surveyed via interviews.<br># community health needs assessment completed with community members.<br># culturally relevant, community educational forums provided.	**Partnership-Precontemplation Phase**<br>Increase consciousness raising by the target population by 70%.<br>Increase environmental reevaluation by the target population by 70%.<br>Increase dramatic relief by the target population by 70%.
**Assessment-Contemplation Phase**<br>Complete community readiness survey with community members.	**Assessment-Contemplation Phase**<br># community readiness survey completed	**Assessment-Contemplation Phase**<br>Increase self-evaluation by the target population by 70% within 12 months.
**Planning-Preparation Phase**<br>Hold an improved cookstove raffle.	**Planning-Preparation Phase**<br># improved cookstoves raffled	**Planning-Preparation Phase**<br>Increase self-liberation by the target population by 70% within 15 months.
		**INTERMEDIATE OUTCOMES**
**Implementation-Action Phase**<br>Conduct baseline survey.<br>Build improved cookstoves.<br>Dispose of traditional, open fire stoves.<br>Substitute cooking method on improved cookstoves.	**Implementation-Action Phase**<br># Baseline surveys conducted<br># improved cookstoves built.<br># traditional, open fire stoves disposed.<br># substituted cooking method on improved cookstoves.	**Implementation-Action Phase**<br>Increase counterconditioning, reinforcement management, and stimulus control by the target population by 40% within 24 months.
**Evaluation-Maintenance Phase**<br>Continue consistent and correct use of improved cookstove.<br>Conduct follow-up survey.	**Evaluation-Maintenance Phase**<br># Continued consistent and correct use of improved cookstove.<br># Follow-up surveys conducted.	**Evaluation-Maintenance Phase**<br>Increase self-efficacy in cooking behaviors by 40% within 36 months.

CONTEXTUAL

- Cultural cooking traditions
- Taste of food
- Cooking time
- Household size
- Income
- Accessibility of cost-effective improved cookstoves
- Affordability of LPG
- Availability of health education
- Acceptability of deforestation
- Availability of electricity
- Affordability of chimneys
- Poorly ventilated kitchens
- Rural environment
- Availability of transportation
- Accessibility of health care

IMPACT

Decrease open fire cooking associated symptoms in the target population by at least 20% within 7 years.
