# Supplementary material for: Improving Health Outcomes in Women Who Use Traditional Open Fire Cookstoves by Addressing Cooking Behaviors: A Longitudinal Cohort Study
Source: Int J Environ Res Public Health. 2026 May 13;23(5):647. doi: 10.3390/ijerph23050647 (PMC13206588; doi:10.3390/ijerph23050647)
Supplement: Supplementary file 1 [file ijerph-23-00647-s001.zip › ijerph-4241338-supplementary final.pdf]

Supplementary Tables

Table S1

Summary of survey instruments, domains, sample items, response format, and study phase of administration.

Instrument	Study Phase	Primary Purpose	Domains Assessed	Sample Items	Response Format	Respondents / Administration
Key Informant Interview Guide	Partnership–Precontemplation Phase	To identify community context, priority health concerns, perceived causes of illness, available resources, and community priorities to inform the community health needs assessment and intervention planning	Regional demographics; community illnesses/injuries; perceived causes and treatment; morbidity/mortality; water; food; sanitation; community health resources; education/health education; transportation/communication; project goals and community priorities	"What are the 5 most important illnesses/injuries affecting people in the community?" "What do people think is the cause of this illness/injury?" "What are your health-related priorities/goals for your community?"	Primarily open-ended interviewer-administered questions; some structured descriptive items	Key informants from the community; interviewer-administered face-to-face with interpreter support as needed
Community Health Needs Assessment Interview Tool	Partnership–Precontemplation Phase	To identify perceived major illnesses, their causes, and community-proposed solutions from	Priority illness/health concern; perceived cause or origin; proposed solutions/resources	"In your opinion, what is the illness that most affects your area?" "What do you consider to be	Open-ended interviewer-administered questions	Community members; face-to-face interviews

		community members		the cause or origin of this illness?” “What proposals (solutions) are presented against these problems?”		
Community Readiness Survey	Assessment–Contemplation Phase	To assess community readiness to address indoor air pollution and support development of the intervention	Community efforts; community knowledge of efforts; leadership; community climate; knowledge about the issue; resources for prevention efforts	“Using a scale from 1–10, how much of a concern is indoor air pollution in your community?” “Please describe the efforts that are available in your community to address indoor air pollution.” “Would the leadership support additional efforts?”	Mixed format: open-ended questions plus anchored 1–10 rating prompts	Community members / local stakeholders; interviewer-administered face-to-face
Baseline Household Survey	Implementation–Action Phase (pre-	To characterize participant households and establish pre-	Household identifiers and family composition; fuel type and fuel use; fuel gathering/buying; fuel drying; literacy/education;	“What types of fuel do you use for cooking?” “Do you ever	Mixed format: categorical response	Primary household cook / participant; interviewer-

	implementation / T0)	intervention measures for fuel use, kitchen environment, smoke exposure, and self-reported health symptoms	occupation; women's and children's health and well-being; smoking exposure; kitchen type; roof/walls/eaves/windows/doors; stove type; smoke extraction; house layout	use 'green' fuel?" "In what ways do you feel that smoke from the fire affects your health?" Prompted symptom items: eyes, cough, chest illness, shortness of breath, headache; "Do other people smoke in the kitchen?"	options, short-answer items, observational items, and open-ended health questions with prompted symptom checklist	administered in the home
Follow-up Household Survey	Evaluation–Maintenance Phase (post-implementation / T1–T4)	To assess post-intervention fuel practices, self-reported symptoms, and sustained/correct use and maintenance of the improved cookstove	Fuel drying; women's and children's health and well-being; smoking exposure; stove-use context; stove satisfaction; perceived wood use; stove maintenance; chimney maintenance; proper use; condition of chimney and plancha	"How do you like your stove?" "How much wood do you use now?" "What did you do to maintain your stove?" Prompted symptom items: eyes, cough, chest illness, shortness of breath, headache;	Mixed format: categorical response options, short-answer items, open-ended questions, and prompted symptom checklist	Primary household cook / participant; interviewer-administered in the home

	“When you are cooking with only one pot of food, what do you do with the other burner while cooking?” “What do you do with both burners after cooking?” “How do you maintain your cookstove? Chimney? Plancha?”	

Note. This table summarizes the formative and household survey instruments used across study phases, including domains, sample items, response formats, and the phase in which each instrument was administered.

Table S2

Characteristics of key informants involved in the partnership–precontemplation phase.

**Characteristic**	**Summary**
Number of key informants	13
Age range	31 to 52 years
Sex	7 male; 6 female
Education	Ranged from 2nd grade to 6 years of college
Occupational background	Health care providers (n = 5)<br>Teachers (n = 2)<br>NGO representatives (n = 2)<br>Faith-based leaders (n = 2)<br>Health promoter (n = 1)<br>Engineer (n = 1)

Note. Key informants were selected to provide a range of community perspectives during the partnership–precontemplation phase.

Table S3

Key informant interview findings: Primary health concerns identified during the partnership-precontemplation phase.

Primary health concern	n	% of total responses
Gastrointestinal illnesses	11	28%
Respiratory illnesses	10	25%
Urinary tract infections	5	13%
Skin infections	5	13%
Hypertension	2	5%
Pregnancy-related concerns	2	5%
Domestic violence	2	5%
Diabetes	1	3%
Arthritis	1	3%
Total	39	100%

Note. Percentages are based on 39 total health-concern responses identified across interviews with 13 key informants. Because key informants could identify more than one concern, totals reflect responses rather than individual participants.

Table S4

Characteristics of community health needs assessment participants.

**Characteristic**	**Summary**
Number of participants	77
Number of households	47
Age range	18 to 90 years
Sex	26 male; 51 female
Education	Ranged from no formal education to high school graduates
Occupational background	Homemakers (n = 47)<br>Farmers (n = 25)<br>Shopkeepers (n = 2)<br>Teacher (n = 1)<br>Student (n = 1)<br>Occupation not specified (n = 1)

Note. Community health needs assessment participants included 77 individuals from 47 households interviewed during the partnership–precontemplation phase.

Table S5

Community health needs assessment findings: Primary health concerns identified during the partnership–precontemplation phase.

Primary health concern	n	% of total responses
Respiratory illnesses	65	35%
Gastrointestinal illnesses	30	16%
Urinary tract infections	24	13%
Fever	17	9%
Arthritis	13	7%
Hypertension	11	6%
Headaches	7	4%
Diabetes	7	4%
Skin infections	6	3%
Anemia	2	1%
Pregnancy-related concerns	2	1%
Dental problems	2	1%
Total	186	100%

Note. Percentages are based on 186 total health-concern responses identified across interviews with 77 community members from 47 households. Because participants could identify more than one concern, totals reflect responses rather than individual participants. Percentages are rounded to the nearest whole number.
